# Research trends and frontiers in lupus nephritis: a bibliometric analysis from 2012 to 2022

**DOI:** 10.1007/s11255-023-03715-w

**Published:** 2023-08-15

**Authors:** Jiaping Qi, Teng Wu, Jing Wang, Ju Zhang, Lin Chen, Zhaoyu Jiang, Yixuan Li, Huan Jiang, Qiong Sun, Qingchen Gu, Zhenhua Ying

**Affiliations:** 1 Center for General Practice Medicine, Department of Rheumatology and Immunology, Zhejiang Provincial People’s Hospital, Affiliated People’s Hospital, Hangzhou Medical College, No. 158, Shangtang Road, Hangzhou, 310014 Zhejiang China; 2 Zhejiang Provincial Key Laboratory of Traditional Chinese Medicine Cultivation for Arthritis Diagnosis and Treatment, Hangzhou, 310000 Zhejiang China; 3 Bengbu Medical College, Bengbu, China; 4 Department of Rheumatology, Affiliated Hospital of Nantong University, Nantong, China; 5 Dalian Medical University, Dalian, China

**Keywords:** Lupus nephritis, Bibliometrics, CiteSpace, VOSviewer, Hotspots

## Abstract

**Objectives:**

Lupus nephritis is a prevalent renal manifestation of systemic lupus erythematosus (SLE) and represents a significant cause of morbidity and mortality associated with the disease. This study endeavors to undertake a meticulous bibliometric analysis of LN publications to comprehend the research hotspots and future directions.

**Methods:**

The literature on LN was acquired from the Web of Science Core Collection (WoSCC). Co-occurrence and cooperative relationship analysis of authors, institutions, countries, journals, references and keywords in the publication was performed through CiteSpace, VOSviewer and a bibliometric online analysis platform. The knowledge graphs were created, and clustering and emergence analyses were performed.

**Results:**

According to the search strategy, a total of 2077 publications related to lupus nephritis (LN) have been identified, with China being the largest contributor globally. The Ohio State University emerged as the most prolific institution. Lupus is the most cited and published journal. Jan J Weening and Brad Rovin were the most prolific and cocited authors. The current research focus revolved around the “nirp3 inflammasome,” “biomarker,” and “voclosporin”. “international society,” “thrombotic microangiopathy (TMA),” and “pathway” were identified to be future research hotpots by keyword burst analysis.

**Conclusions:**

This bibliometric analysis summarizes for the first time the progress of LN research (2012–2022), and qualitatively and quantitatively evaluates the bibliometric information of LN research. There has been a steady increase in the scientific literature on LN over the past 11 years, with an average growth rate of 7.27%. In this field, researchers are primarily based in China and the United States. The pathogenic mechanisms, management strategies and prognostic outcomes of LN are acknowledged as prospective research hotspots. Bibliometrically, the research status and trends of LN publications may greatly assist and be a significant reference for future research in the area.

## Introduction

Lupus nephritis (LN) is a severe form of glomerulonephritis and the primary manifestation of systemic lupus erythematosus (SLE), an autoimmune disease. LN usually appears within 5 years after the diagnosis of SLE and can proceed to end-stage kidney disease (ESKD) in 5–20% of patients within 10 years [1–4]. SLE incidence ranges from 1.5 to 11 per 100,000 person-years and prevalence from 13 to 7713.5 per 100,000 individuals [5]. The gold standard for LN diagnosis is renal biopsy, which can classify renal involvement into six categories [6]. According to the Kidney Disease Improving Global Outcomes (KDIGO) programme, CKD is diagnosed when problems in kidney structure or function last longer than 3 months. Patients with class III, IV, or V LN are at imminent risk of developing chronic kidney disease (CKD). Class VI essentially corresponds to renal atrophy in end-stage kidney disease (ESKD) [7–9]. Glucocorticoids (GCs) and other immunosuppressive agents, such as cyclophosphamide (CPA), mycophenolate mofetil (MMF) and calcineurin inhibitors (CNIs), are widely used in standard induction treatment for LN [10]. However, LN has a poor 20–30% remission rate after 6 months of conventional therapy. In addition, GCs-induced side effects further compromise the prognosis of LN [6]. It is anticipated that biological medicines that target the inflammatory process and the autoimmune response would bring hope for refractory LN.

Novel technology and diagnostic standards update LN research hotspots and trends. LN researchers from multiple countries have produced many articles. The literature on LN has grown, but clear summaries are still scarce. Manually studying the literature makes it difficult to portray the study subject's whole picture and growth. Bibliometric analysis a statistical analysis method that assesses and monitors the progress of a particular discipline by analyzing published data [11]. It can display the output and citations of countries, institutions, and authors. In addition, it can identify the keywords in hotspots and research frontiers [12]. Relevant literature is exploding. A systematic overview and summarization of LN from a bibliometric perspective are necessary to benefit research participants.

This paper conducted an 11-year (2012–2022) longitudinal analysis using bibliometric analysis techniques. The following criteria were used to analyze the published literature: year of publication, country/region, institution, author, cocited authors, journal, cocited journals, citations, cocited references and keywords. Finally, a bibliometrics-guided conventional review is performed to describe the research hotspots and future trends. This is the first attempt to conduct intensive statistical analyses of LN-related publications worldwide, which can provide a reference for future research on LN.

## Materials and methods

### Data collection

**Fig. 1:**
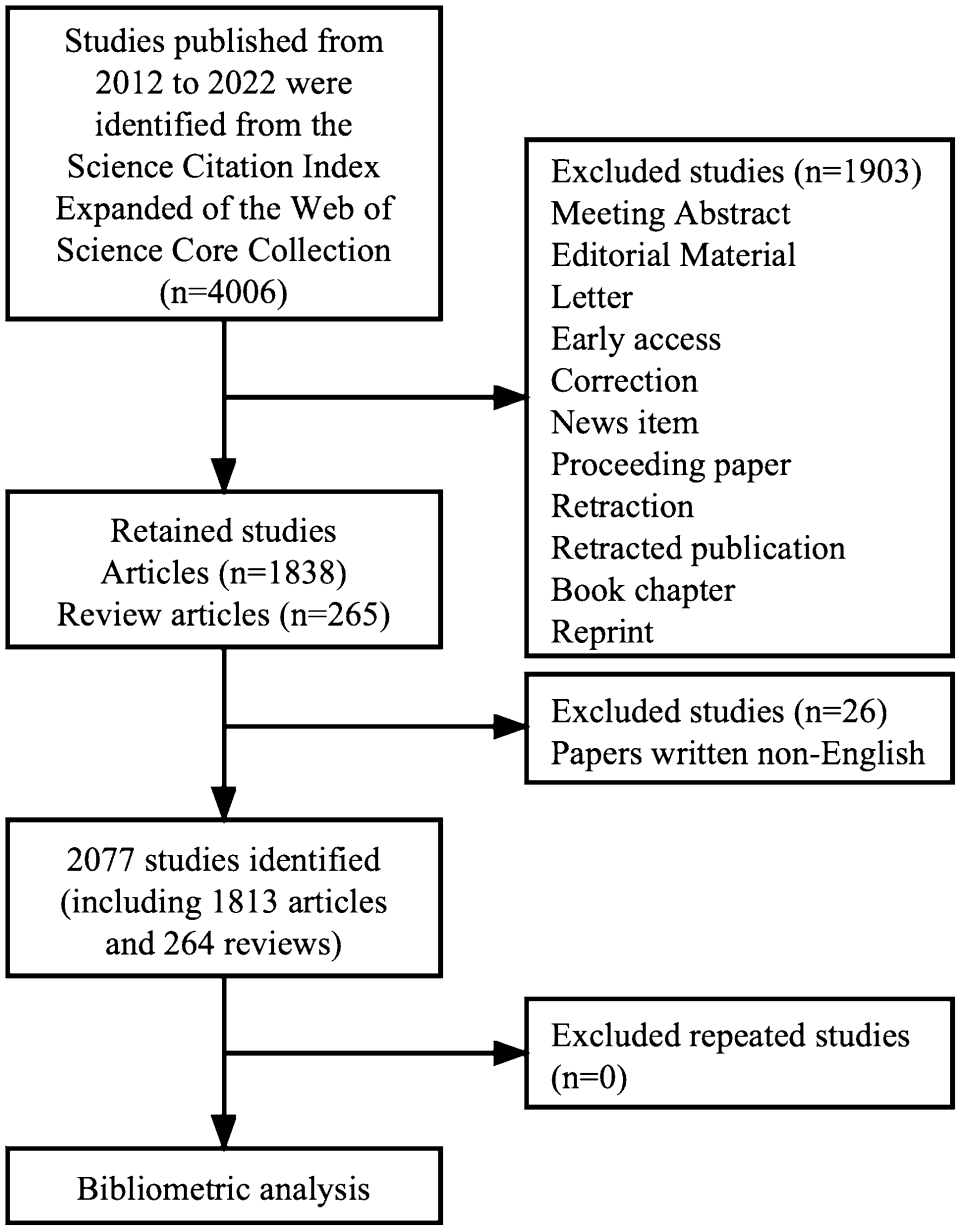
Flowchart of study retrieval and selection

With over 12,000 prestigious and high-quality academic periodicals, Web of Science (WoS) is a reputable and extensive database that provides access to international academic sources [13]. All documents published between 2012 and 2022 were retrieved and downloaded from the Science Citation Index Expanded (SCI-Expanded) of the WoS Core Collection (WoS CC) database on 3 December 2022. To confirm that the data were highly relevant, “Lupus nephritis”-related terms were searched by title (TI). The retrieval formula was as follows: TI = (Lupus nephritis OR Lupus Glomerulonephritis OR Nephritis, Lupus OR Lupus Nephritides OR Nephritides, Lupus OR Glomerulonephritis, Lupus OR Glomerulonephritides, Lupus OR Lupus Glomerulonephritides). The time frame was restricted to the years 2012 to 2022. The study only focused on reviews and articles written in English. Ultimately, 2077 documents were obtained and saved in plain text. Finally, CiteSpace software was used to deduplicate these papers. Figure 1 shows the detailed retrieval process and screening steps. The primary data search was carried out independently by JPQ and TW, who then discussed any inconsistencies. The ultimate agreement score was 0.90, which denotes a high degree of agreement.

### Bibliometric analysis

The number of annual publications related to LN was analyzed using a line chart created with Microsoft Excel 2019. In addition, three bibliometric tools were employed to conduct comprehensive bibliometric analyses. CiteSpace (version 6.1. R6), Java-based bibliometric software, was utilized to visualize the knowledge framework and evaluate scientific publications. Knowledge maps intuitively illustrate research hotspots and future development tendencies [14]. The collaboration of organizations and writers was carried out using CiteSpace. Cocitation analysis was used to identify burst references and keywords. The explanations put out by CiteSpace comprised the following: timespan: 2012–2022 (slice length = 1), selection criteria: g-index (*k* = 25), pruning: pathfinder, pruning sliced networks, and pruning the merged network. VOSviewer (version 1.6. 18) is a program for building and viewing bibliometric maps that has been successfully used in a variety of projects undertaken by scientific and technical research centers and can display large bibliometric maps in an easy-to-interpret manner [15]. Furthermore, VOSviewer offers three distinct perspectives for the visualization of co-occurrence and citation networks in the scientific literature: the density view, cluster density view, and scatter view [15]. VOSviewer software was utilized in the present study to conduct a comprehensive co-occurrence analysis of keywords, citations, and cocitation relationships among scientific journals. This methodology allowed for the identification of critical publications and research trends in the field. In addition, a bibliometric online analysis tool (https://bibliometric.com/) was employed to assess the coauthorship patterns among countries.

## Results

### Publishing trend analysis

**Fig. 2:**
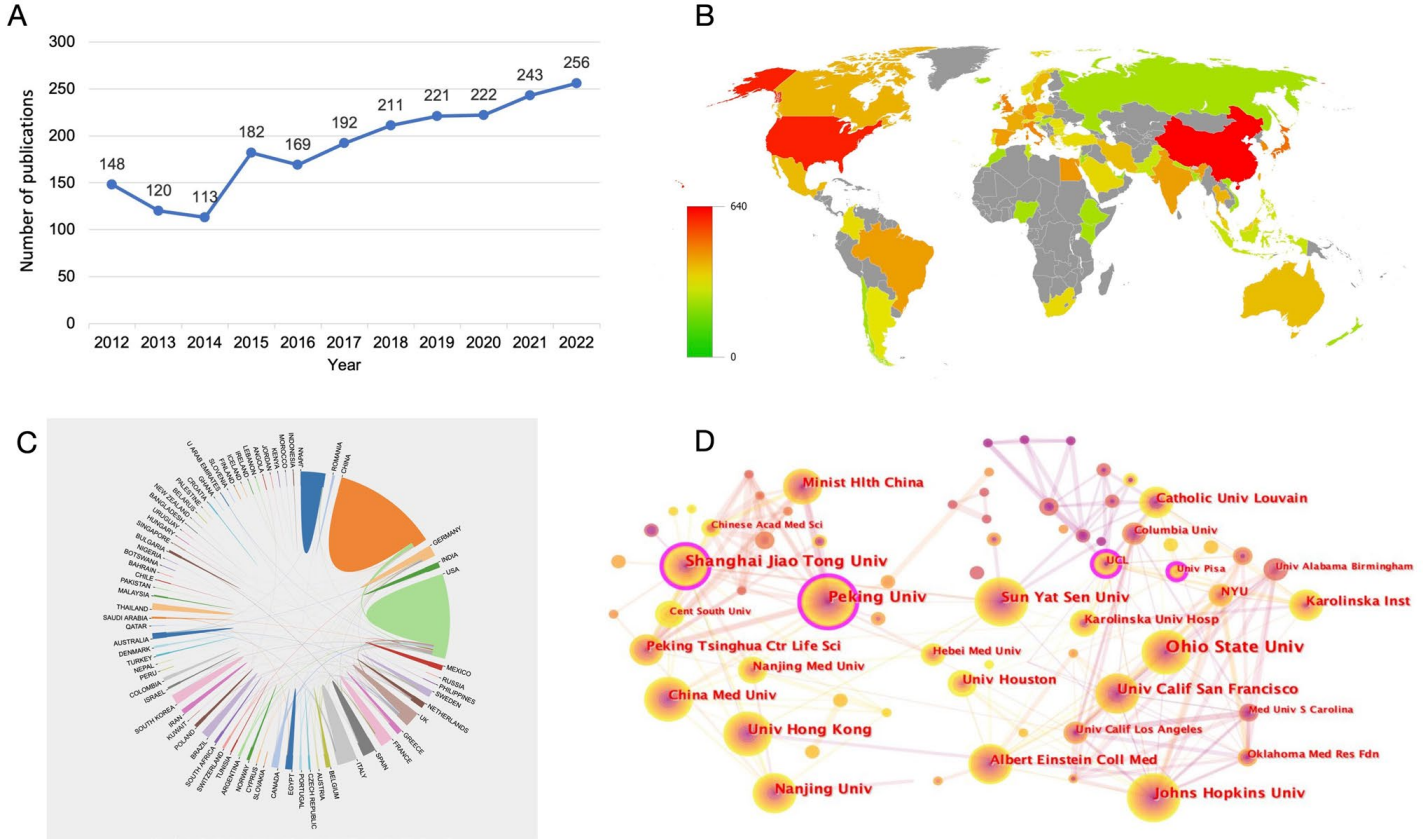
**A** Lupus nephritis (LN) annual outputs from 2012 to 2022. **B** Map of the source distribution of LN publications. **C** International cooperation between countries is analyzed through an online analytics platform. The curve between countries reflects the closeness of cooperation, and thicker lines indicate closer cooperation. **D** CiteSpace interinstitutional international collaboration analysis. Each circle represents an institution, and the size of the circle is proportional to the number of publications. Circles with higher centrality (> 0.1) are indicated by purple rings. The lines indicate the strength of the collaborative relationship, with thicker lines indicating stronger collaboration. The line color represents the time of the first coauthorship, with a more yellow color indicating closer to 2022 and a redder color indicating closer to 2012

After the removal of duplicate publications using CiteSpace software, 2077 publications relating to LN were identified from the SCI-Expanded database of WoSCC. Figure 2A illustrates the annual LN publication distribution and highlights two research trends. From 2012 to 2014, the publication rate steadily declined, reaching a nadir in 2014(*n* = 113). However, from 2014 to 2021, the number of publications increased quickly, peaking in 2022(*n* = 256). Notably, the volume of publications in 2022 was 2.26 times greater than that in 2014.

### Visualization of country collaboration by a bibliometric online analysis platform

Figure 2B depicts Bibliometric’s global output map. A total of 371 institutions from 64 countries contributed to the 2077 identified documents. Table 1 highlights the top ten countries in LN research, with China (632, 30.43%), the United States (411, 19.79%) and Japan (118, 5.68%) comprising the top three. Without considering each country's demographics, a ratio indication of published papers per million people was used. South Korea led with 1.29 articles per million after population adjustment. Regarding centrality, the top three countries were the United Kingdom (0.23), China (0.14) and the United States (0.14). The collaboration relationships among countries are presented in Fig. 2C, with the width of the line indicating the frequency of cooperation and the thicker line indicating tighter cooperation. Notably, China and the United States, which together create 1043 pieces of literature (50.22%), collaborate frequently.

**Table 1:** Top ten countries with the highest productivity related to Lupus nephritis (LN)

Rank	Country	Count (% of 2077)	Number of papers per million people	Centrality
1	China	632 (30.43)	0.45	0.14
2	USA	411 (19.79)	1.24	0.14
3	Japan	118 (5.68)	0.94	0.08
4	UK	76 (3.66)	1.13	0.23
5	Italy	69 (3.32)	1.17	0.10
6	South Korea	67 (3.23)	1.29	0.01
7	Germany	54 (2.6)	0.65	0.02
8	Egypt	50 (2.41)	0.48	0.01
9	Spain	48 (2.31)	1.01	0.04
10	Brazil	45 (2.17)	0.21	0.06

Rank: based on the number of publications. The World Bank's official website (https://data.worldbank.org.cn) provided the demographic data

### Visualization of institutional collaboration by CiteSpace

Six of the top ten most prolific institutions were Chinese, while four were American (Table 2). The Ohio State University emerged as the most productive institution (74, 3.56%), followed by Peking University (61, 2.94%) and Shanghai Jiao Tong University (52, 2.50%). Figure 2D displays a node-link diagram in which each node denotes an institution, and its size represents the number of publications produced by the institution. Circles with a high centrality (> 0.1) are indicated by purple rings. Shanghai Jiao Tong University ranked first in centrality with a score of 0.14, followed by Peking University (0.13) and the University College London (0.12). The figure also shows that Peking University and The Ohio State University are leading research on LN in China and the United States, respectively.

**Table 2:** Top ten most productive institutions related to LN

Rank	Institution	Count (% of 2077)	Centrality
1	Ohio State Univ (USA)	74 (3.56)	0.08
2	Peking Univ (China)	61 (2.94)	0.13
3	Shanghai Jiao Tong Univ (China)	52 (2.50)	0.14
4	Sun Yat Sen Univ (China)	46 (2.21)	0.09
5	Nanjing Univ (China)	43 (2.07)	0.04
6	Univ Hong Kong (China)	43 (2.07)	0.02
7	Johns Hopkins Univ (USA)	42 (2.02)	0.05
8	Univ Calif San Francisco (USA)	35 (1.69)	0.05
9	Albert Einstein Coll Med (USA)	31 (1.49)	0.04
10	China Med Univ (China)	30 (1.44)	0.07

Rank: based on the publication count

### Visualization of authors and cocited authors by CiteSpace

**Fig. 3:**
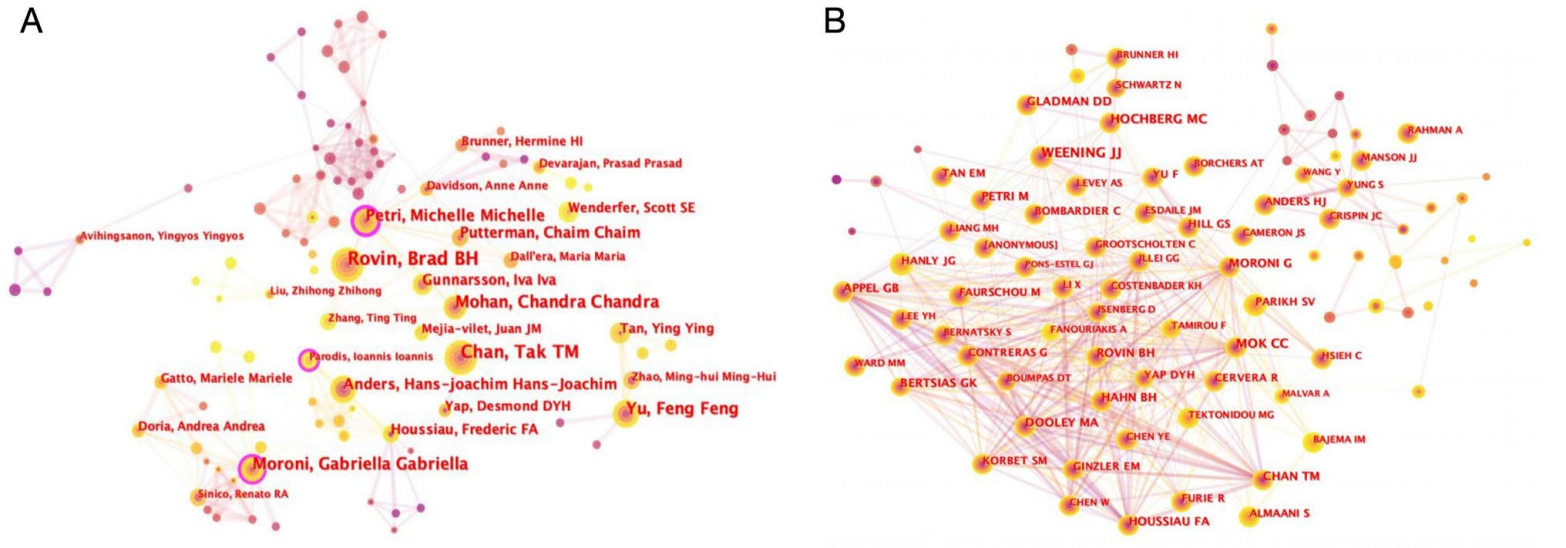
**A** Map of authors in LN research from CiteSpace. **B** Representation of cocited authors who are active in LN using CiteSpace. Each circle represents a different author, and the size of the circle is related to the number of publications (A) or citations (B). The purple bands around the circles indicate those with a greater centrality (> 0.1). The thickness of the lines indicates the level of coauthorship (A) or cocitation (B), with larger lines indicating higher levels of collaboration (A) or relevance (B). The line color indicates when the first coauthorship occurred, with a more yellowish hue indicating a time closer to 2022 and a redder hue indicating a time closer to 2012

492 authors were identified from 2077 LN-related articles. The most productive authors are identified in Table 3, with Brad Rovin being the most prolific author (44, 2.12%), followed by Tak Chan (35, 1.69%) and Chandra Mohan (30, 1.44%). The degree of collaboration among authors is depicted in Fig. 3A, with Michelle Petri (0.27), Ioannis Parodis (0.16), and Gabriella Moroni (0.15), who have purple outer circles, identified as the most central authors, playing crucial bridging roles in LN research.

**Table 3:** Top ten authors with the most publications involved in LN

Rank	Author	Count (% of 2077)	Centrality
1	Brad Rovin	44 (2.12)	0.08
2	Takmao Chan	35 (1.69)	0.09
3	Chandra Mohan	30 (1.44)	0.07
4	Feng Yu	29 (1.40)	0.01
5	Gabriella Moroni	28 (1.35)	0.15
6	Michelle Petri	25 (1.20)	0.27
7	Hans-joachim Anders	20 (0.96)	0.02
8	Chaim Putterman	19 (0.91)	0.01
9	Frederic Houssiau	15 (0.72)	0.04
10	Iva Gunnarsson	15 (0.72)	0.01

Rank: based on the publication count

The simultaneous citation of two writers by a third work is recognized as cocitation. The cocitation analysis of LN research has identified the top ten most frequently cited authors (Table 4), with Weening JJ being the most cocited (809), followed by Hochberg MC (527) and Austin HA (496). Figure 3B displays the cocitation relationships between cocited authors, with Hahn BH being the most frequently cocited author with a centrality of 0.06. Hahn BH is also the most prolific author, which indicates that his research is rich and recognized by other researchers in the field.

**Table 4:** Top ten co-cited authors with the most citations involved in LN

Rank	Author	Count (% of 2077)	Centrality
1	Weening JJ	809 (38.95)	0.01
2	Hochberg MC	527 (25.37)	0
3	Austin HA	496 (23.88)	0.02
4	Mok CC	482 (23.21)	0.02
5	Moroni G	400 (19.26)	0.01
6	Rovin BH	391 (18.83)	0.06
7	Hahn BH	389 (18.73)	0.02
8	Houssiau FA	379 (18.25)	0.01
9	Appel GB	357 (17.19)	0.01
10	Petri M	332 (15.98)	0.01

Rank: based on the publication count

### Visualization of journals and cocited journals by VOSviewer

**Fig. 4:**
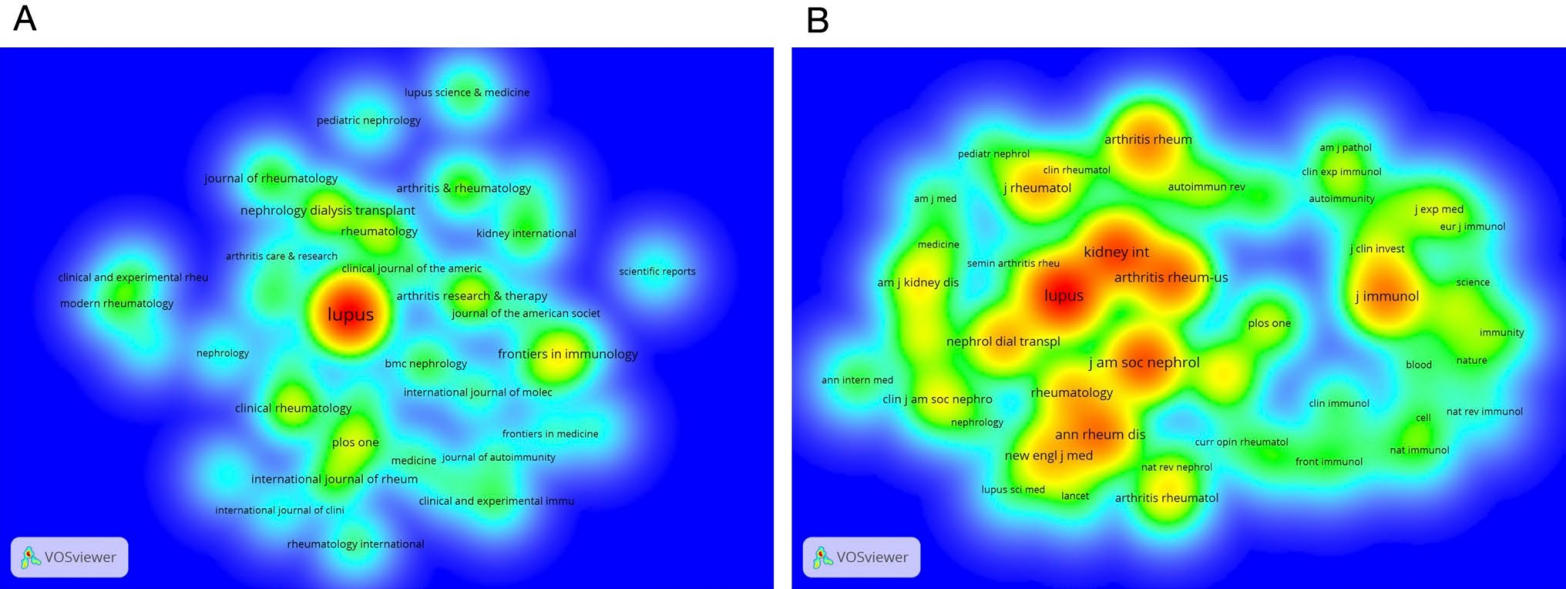
**A** Density map produced by VOSviewer for journals with at least 15 LN research papers. The size of the title of the journal is proportional to the publication output. The bottom background color becomes closer to red as the journal publishes more articles. **B** Density map created by VOSviewer of cocited journals with LN citations totaling less than 300. The number of citations is proportional to the size of the journal title. The bottom background color becomes closer to red when a journal acquires more citations

The present study surveyed 395 academic journals that published articles on LN research and identified the top ten most productive journals, contributing 32.06% (666). Lupus ranked first in terms of the number of publications published (267, 12.86%), followed by Frontiers Immunology (56, 2.70%) and Nephrology Dialysis Transplantation (50, 2.41%), as reported in Table 5. Figure 4A presents a density map of the journals with ≥ 15 publications. Among the top five periodicals, five journals were ranked Q1 in the Journal Citation Reports (JCR) 2021 standards, with Arthritis and Rheumatology boasting the highest impact factor (IF; 15.483). In conclusion, the contributions of these ten journals offer a solid framework for further study in LN.

**Table 5:** Top ten most productive journals related to LN

Rank	Journal	Count (% of 2077)	IF(2021)	JCR(2021)
1	Lupus (the United Kingdom)	267 (12.86)	2.858	Q4
2	Frontiers In Immunology (Switzerland)	56 (2.70)	8.786	Q1
3	Nephrology Dialysis Transplantation (the United Kingdom)	50 (2.41)	7.186	Q1
4	Arthritis Research and Therapy (the United Kingdom)	50 (2.41)	5.606	Q1
5	Clinical Rheumatology (the United Kingdom)	48 (2.31)	3.650	Q3
6	Plos One (the United States)	47 (2.26)	3.752	Q2
7	Rheumatology (the United Kingdom)	41 (1.97)	7.046	Q1
8	Arthritis and Rheumatology (the United States)	38 (1.83)	15.483	Q1
9	International Journal Of Rheumatic Diseases (Australia)	35 (1.69)	2.558	Q4
10	Journal Of Rheumatology (Canada)	34 (1.64)	5.346	Q2

*Rank* based on the publication count,* IF* impact factor,* JCR* journal citation reports

Table 6 presents the cocitation frequency of journals related to LN, and it reveals that Lupus is the most cited journal (5430), followed by Kidney International (3971) and Journal of the American Society of Nephrology (3687). Eight of the top ten cocited journals have citation frequencies exceeding 2000. A density map displays the cocited journals with citation frequencies of at least 300 (Fig. 4B). Furthermore, among these cocited periodicals, Annals of the Rheumatic Diseases has the highest impact factor (28.003), followed by Kidney International (18.998) and Arthritis and Rheumatology (15.483).

**Table 6:** Top ten co-cited journals with the most citations related to LN

Rank	Co-cited journal	Citation	IF (2021)	JCR (2021)
1	Lupus	5430	2.858	Q4
2	kidney international	3971	18.998	Q1
3	Journal Of The American Society Of Nephrology	3687	14.981	Q1
4	Arthritis And Rheumatism	3297	NA	NA
5	Journal of Immunology	2867	5.430	Q2
6	Annals Of The Rheumatic Diseases	2864	28.003	Q1
7	Arthritis and Rheumatology	2432	15.483	Q1
8	Nephrology Dialysis Transplantation	2098	7.186	Q1
9	Journal Of Rheumatology	1916	5.346	Q2
10	Rheumatology	1809	7.046	Q1

Rank: based on the citation count

### Analysis of high-citation publications

Examining high-citation publications provides valuable insights into the fundamental research of a discipline. Table 7 lists the top ten publications on LN. Three of them are original articles, while seven were reviews that were all written between 2012 and 2020. They have each received more than 80 citations in total. The paper that received the most references is Almaani et al. [16] in the Clinical Journal of the American Society of Nephrology (175), followed by Bajema et al. [17] in Kidney International (142) and Hahn et al. [18] in Arthritis Care and Research (141). These studies are widely recognized as the knowledge foundation for research on LN.

**Table 7:** Top ten most cited publications on LN

Rank	Title	First author	Journal	Years	Total citation
1	Update on Lupus Nephritis	Almaani, S	Clinical Journal of the American Society of Nephrology	2017	175
2	Revision of the International Society of Nephrology/Renal Pathology Society classification for lupus nephritis: clarification of definitions, and modified National Institutes of Health activity and chronicity indices	Bajema, IM	Kidney international	2018	142
3	American College of Rheumatology guidelines for screening, treatment, and management of lupus nephritis	Hahn, BH	Arthritis Care and Research	2012	141
4	Joint European League Against Rheumatism and European Renal Association–European Dialysis and Transplant Association (EULAR/ERA–EDTA) recommendations for the management of adult and paediatric lupus nephritis	Bertsias, GK	Annals Of The Rheumatic Diseases	2012	122
5	The frequency and outcome of lupus nephritis: results from an international inception cohort study	Hanly, JG	Rheumatology	2016	104
6	Lupus nephritis	Anders, HJ	Nature Reviews Disease Primers	2020	93
7	Mycophenolate versus azathioprine as maintenance therapy for lupus nephritis	Dooley, MA	New England Journal Of Medicine	2011	89
8	2019 Update of the Joint European League Against Rheumatism and European Renal Association–European Dialysis and Transplant Association (EULAR/ERA–EDTA) recommendations for the management of lupus nephritis	Fanouriakis, A	Annals Of The Rheumatic Diseases	2020	88
9	Risk of End-Stage Renal Disease in Patients With Lupus Nephritis, 1971–2015: A Systematic Review and Bayesian Meta-Analysis	Tektonidou, MG	Arthritis and Rheuma + B10tology	2016	87
10	Efficacy and safety of rituximab in patients with active proliferative lupus nephritis: the Lupus Nephritis Assessment with Rituximab study	Rovin, BH	Arthritis And Rheumatism	2012	86

Rank: based on the citation count

### Visualization of cocited references and references burst by Citespace

**Fig. 5:**
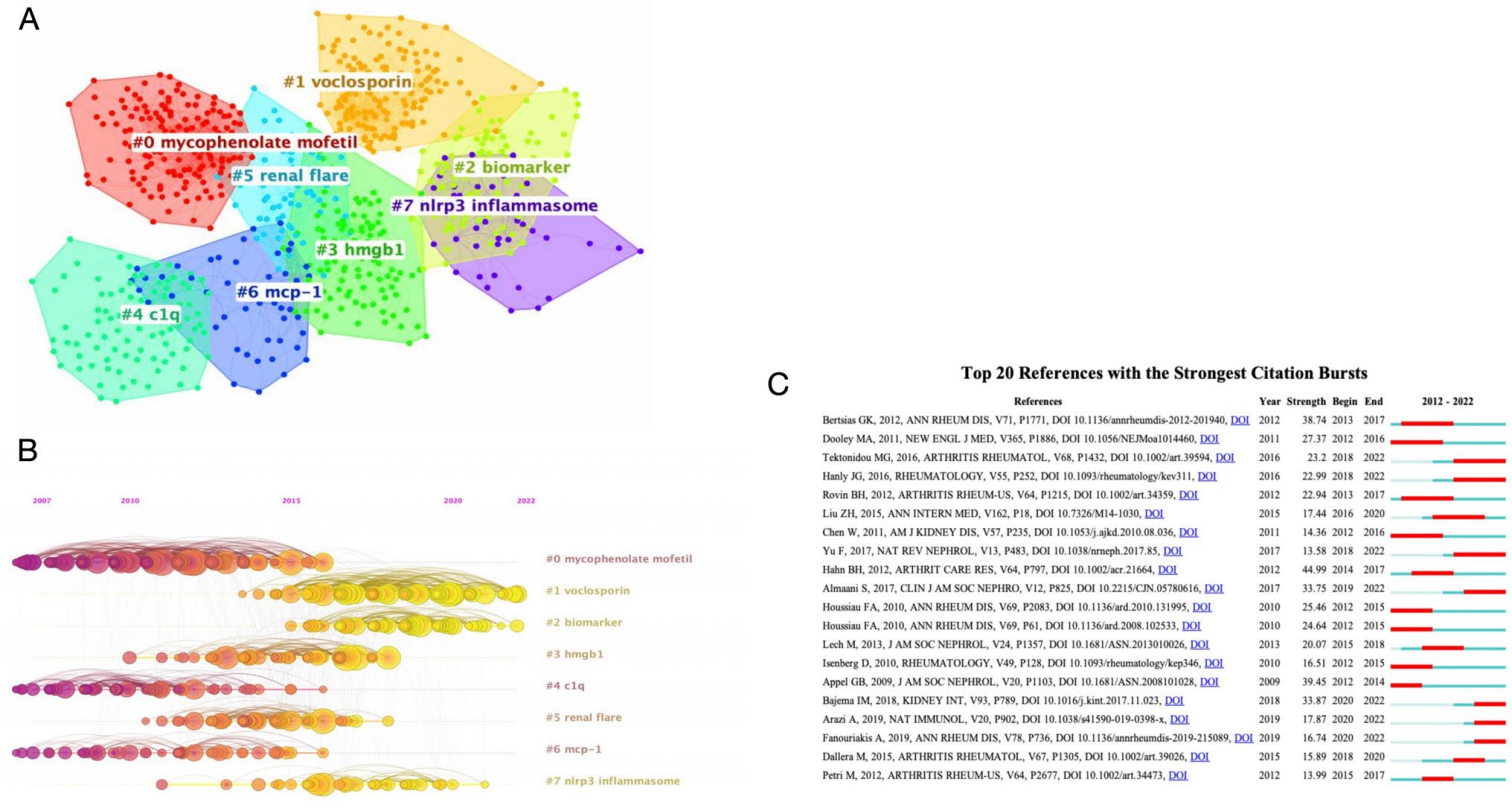
**A** Ten cluster maps based on CiteSpace's literature cocitation analysis. Each cluster is represented by a different color, and the smaller the numerical label, the larger the cluster origin and the more cocited literature (points) it contains. **B** Map of the timeline view of literature cocitation analysis generated by CiteSpace. The elements on the horizontal axis indicate the cocited literature; the position of the elements on the horizontal axis indicates the time of first occurrence; and the connecting lines between the elements indicate the cocitation relationship. The size of the elements is proportional to the number of references cited. The more yellow the color, the closer to 2021, and the more red the color, the closer to 2012. **C** Top 20 references of the strongest CiteSpace citation bursts. The red segments denote the length of the reference breakout, and the green lines denote the time span of these cocited references

Cocitation studies are an important method for investigating the development and identifying the frontiers of a particular field. This study used CiteSpace to analyse cocited references and create a network map with seven clusters using the likelihood ratio (LLR) method. A mean silhouette (S) value greater than 0.7 suggested strong clustering results. A modularity (Q) score above 0.3 indicated strong node grouping. The results of this investigation were reliable, as evidenced by the Q value of 0.6164 and the S value of 0.8662. A separate color symbolized each cluster, with a smaller number labeling a larger cluster profile. Table 8 illustrates that the largest cluster was “mycophenolate mofetil,” (#0), followed by “voclosporin” (#1), “biomarker” (#2) and “High Mobility Group Protein B1 (hmgb1)” (#3). The temporal progression of each cluster can be observed through the timeline view presented in Fig. 5B, whereby the time of the first instance of each element is depicted on the horizontal axis. The size of the element was proportional to the citation count of the reference, and the line connecting the elements denoted the cocited relationship. A yellow color indicated a closer proximity to 2022, while a purple color indicated a closer proximity to 2012. Research hotspots shifted from “mycophenolate mofetil” (#0), “Complement C1q (c1q)” (#4), and “monocyte chemoattractant protein-1 (mcp-1)” (#6) to “nirp3 inflammasome” (#7), “biomarker” (#2), and "voclosporin" (#1).

**Table 8:** Clusters information of co-cited references on LN

Cluster ID	Size	Silhouette	Label (LLR)	Mean years
#0	97	0.877	Mycophenolate mofetil	2010
#1	87	0.89	Voclosporin	2018
#2	56	0.865	Biomarker	2018
#3	48	0.753	hmgb1	2015
#4	52	0.858	c1q	2010
#5	50	0.815	Renal flare	2014
#6	37	0.958	mcp-1	2011
#7	30	0.892	nlrp3 inflammasome	2017
#8	29	0.918	Rituximab	2009

Rank: based on the size. “Size” denotes the amount of co-cited references that a cluster contains. LLR: log-likelihood ratio

Burst detection can detect sudden spikes in reference or phrase prevalence, indicating a considerable surge in attention to a given issue at a specific period. Figure 5C demonstrates the top twenty references with the highest citation bursts, with red segments denoting the length of the reference breakout and green lines denoting the time span of these references. Hahn et al. [18] in Arthritis Care and Research had the strongest outburst (44.99), followed by Appel et al. [19] in the Journal of the American Society of Nephrology (39.45) and Bertsias et al. [20] in Annals of the Rheumatic Diseases (38.73). There were 19 authors contributed to the top 20 articles, although most of the literature outbreaks have subsided, five references are still experiencing citation outbreaks, representing areas of future research focus.

### Visualization of keyword co-occurrence and evolution by VOSviewer and visualization of keyword bursts by CiteSpace

**Fig. 6:**
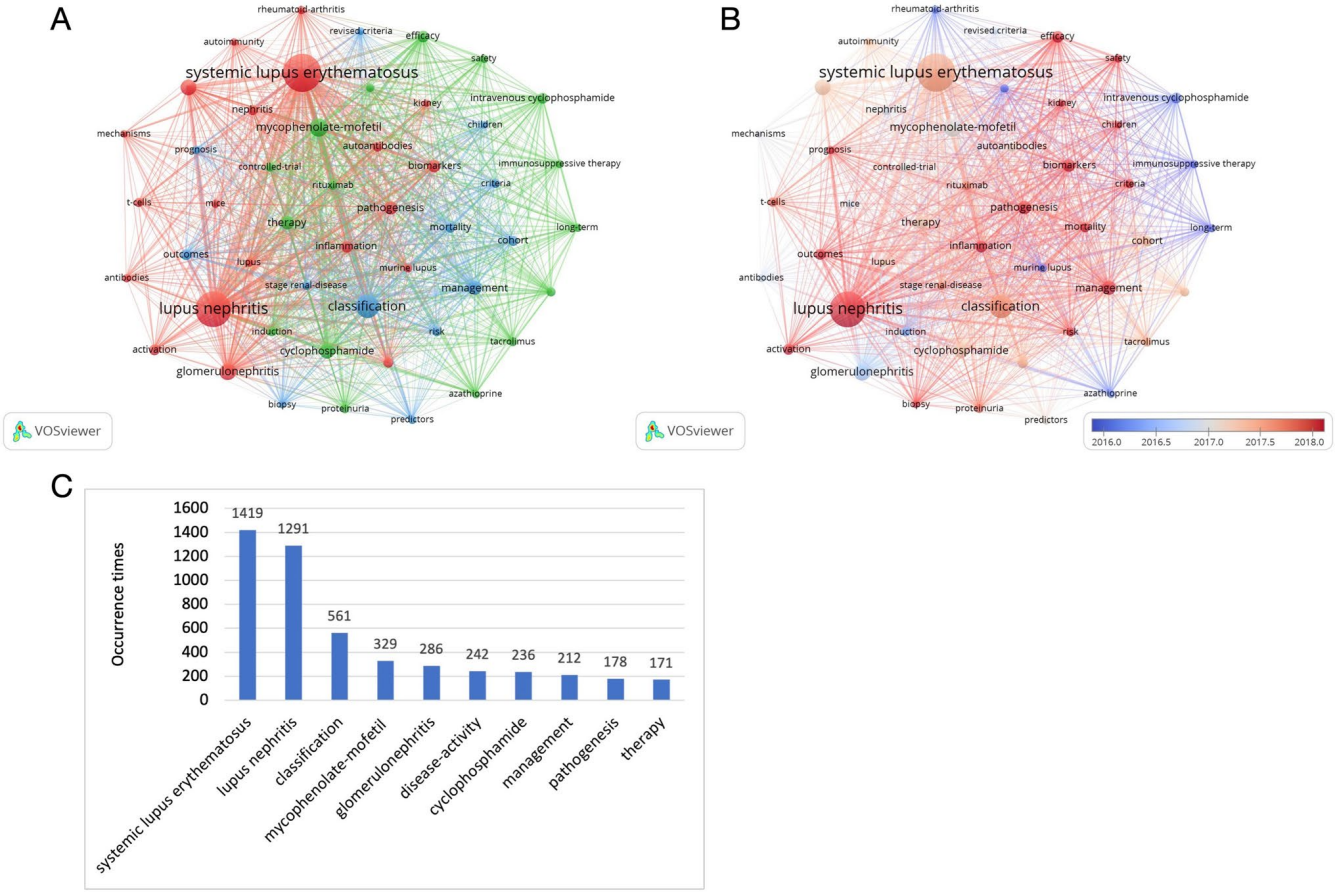
**A** Network clustering map based on VOSviewer's co-occurrence analysis of terms. The number of keyword frequencies must meet a minimum standard of 60. Five clusters of keywords were created: Cluster 1 (red), Cluster 2 (green), Cluster 3, and Cluster 4. (blue). The size of each node, which represents a keyword, is inversely correlated with its frequency. The relationship between two nodes is shown by the line between them. The degree of relevancy is indicated by the distance between nodes; the closer the distance is, the higher the degree of relevancy. **B** Map overlay of a co-occurrence analysis of terms using VOSviewer. According to the color gradient in the lower right corner, the node color indicates the matching average occurrence time. Keywords that emerge relatively early are represented by nodes in blue, whereas keywords that appear later are represented by nodes in red. **C** Top ten words with the most occurrences

Co-occurrence analysis of keywords is an effective means of identifying the predominant themes in a given field. In this study, 49 keywords were merged based on their content, resulting in three distinct clusters (minimum frequency of a keyword ≥ 60) as determined through clustering analysis. Figure 6A depicts each keyword as a node, with the diameter of the node indicating how frequently the term occurs. The distance between nodes shows the significance level, while the lines linking the nodes indicate co-occurrence associations. The three clusters were classified by color, with SLE, MMF, and classification as central themes. Nodes on similar topics are grouped into the same color-coded cluster, consisting of studies on pathogenesis and manifestations (Cluster 1, represented in red), studies on treatment (Cluster 2, represented in green), and studies on classification and outcome (Cluster 3, represented in blue).

Figure 6B presents a visualization of the temporal evolution of keywords, with the keywords that appeared earlier depicted in blue and those that emerged later in red. The earlier period was characterized by topics, such as “cyclophosphamide,” “immunosuppressive therapy,” “murine lupus,” “rheumatoid arthritis,” and “azathioprine syndrome.” In contrast, more recent keywords such as “pathogenesis,” “management,” “safety,” “biomarkers,” and “criteria” suggest that these topics are currently receiving much attention.

**Fig. 7:**
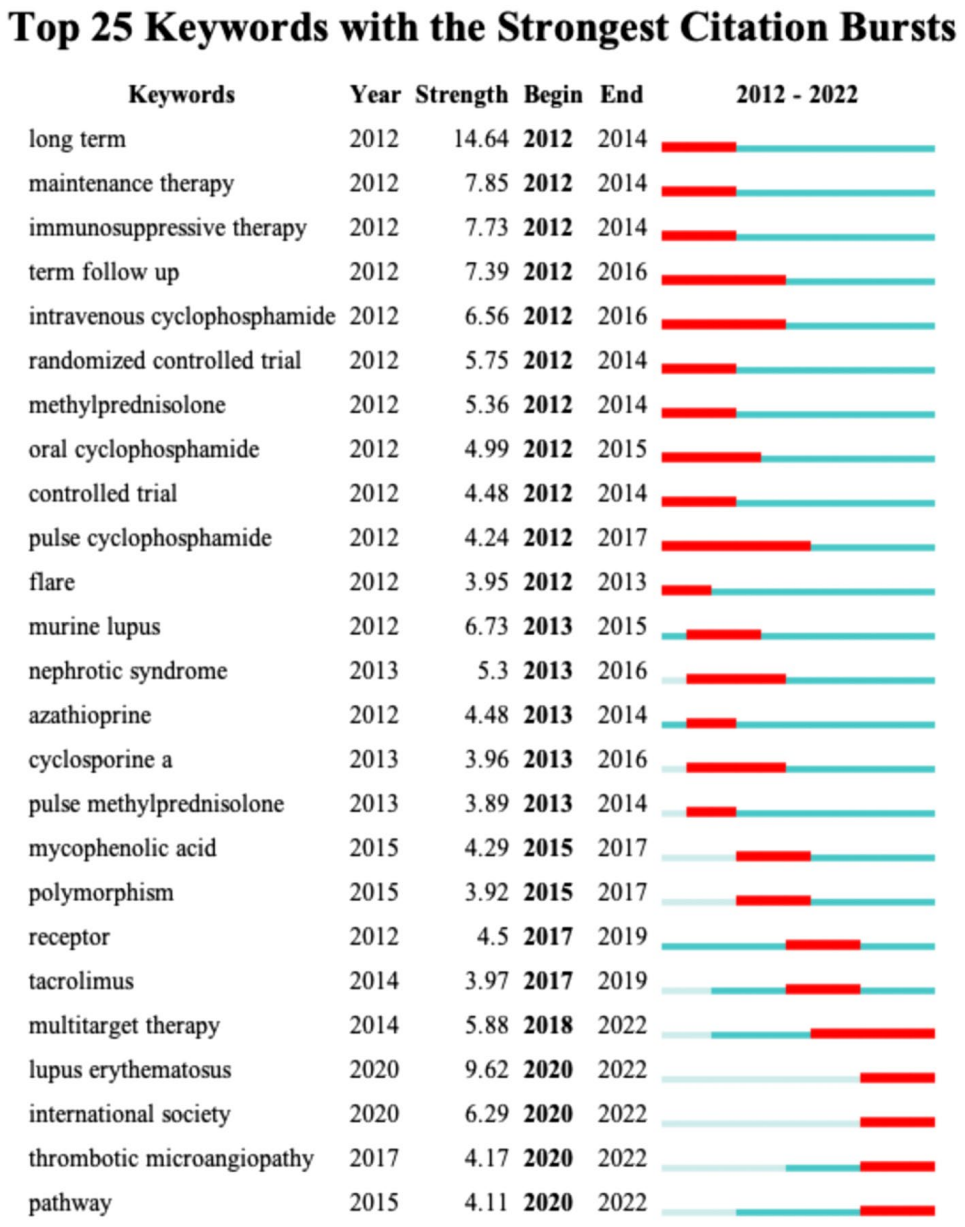
Top 25 keywords with the strongest citation bursts by CiteSpace. Green segments represent the time, and red segments represent the length of the burst from beginning to end

A total of 10 words occur more than 170 times, according to Fig. 6C. SLE (1419) was the most frequent keyword, followed by LN (1291), classification (561), MMF (329) and glomerulonephritis (286). The top 25 burst keywords are shown in Fig. 7. The terms “multitarget therapy,” “lupus erythematosus,” “international society,” “TMA,” and “route” are still in the epidemic phase, spanning the years 2014 to the present.

## Discussion

### Examination of hotspots and frontiers

Reference cocitation analysis is a practical tool for assessing development and locating hotspots within a particular field. Table 8 demonstrates that prior to 2012, “rituximab” (#8), “mycophenolate mofetil” (#0), “c1q” (#4), and “mcp-1” (#6) were the themes that appeared most frequently. Voclosporin (#1) and “biomarker” (#2) are the most popular terms after 2017, indicating that the problems in the clusters mentioned above are the current research priorities.

Voclosporin is an oral calcineurin inhibitor immunosuppressant that can be used in combination with other immunosuppressants to treat active adult LN and is the first oral medication approved in the United States for this indication. Compared to cyclosporine, a previous-generation calcineurin inhibitor, it is superior in improving the potency and metabolic stability of calcium-regulated phosphatase inhibition [21], blocking lymphocyte proliferation and the T-cell-mediated immune response, maintaining the integrity of the actin cytoskeleton of podocytes, and reducing proteinuria [22]. A real-world study demonstrated better efficacy with voclosporin compared to a placebo combined with MMF and a low-dose steroid. The combined findings from phase 2 and phase 3 clinical studies on voclosporin mark a significant improvement in the management of patients with active LN [23].

Over the past decades, biomarkers have been widely screened, and multiple variants associated with LN have been identified at the genomic, transcriptomic and proteomic levels [24]. Some biomarkers of relatively high research interest are hmgb1, c1q, mcp-1 and the NOD-like receptor thermal protein domain associated protein 3(nlrp3) inflammasome. These studies and findings can help identify potential therapeutic targets for early treatment to improve outcomes.

The evolutionary path of each cluster is shown in the cocited literature's timeline view (Fig. 5B). Citation explosion refers to an abrupt increase in citations for a specific publication at a particular stage. It can be a valuable tool to spot developing popular subjects at a specific time. Figure 5C displays the top twenty publications with the highest citation explosion. Seven documents (35%) in the top twenty are still in a bursting condition and focus on the diagnosis and management of LN, indicating that these research topics continue to dominate LN research.

The study by Bajema et al. [17] in Kidney International in 2017 has the strongest explosive intensity (33.87) among the seven papers above. The consensus report on the definition and classification of glomerular lesions in LN. Mesangial hypercellularity and cellular, fibrouscellular and fibrous crescents are given new definitions. In addition, the report excluded the IV-S and IV-G subdivisions for Class IV LN, arguing that the active and chronic names for Class III/IV lesions were replaced by active and chronic indices that apply to all grades. Almaani et al. [16] offered the article in second place (33.75), in which it was discovered that understanding the disease has yet to result in significant therapeutic advancements using a summary of the most current understanding of disease epidemiology, genetics, pathophysiology, and treatment. Nonetheless, the availability of various cutting-edge immunosuppressive medications and a well-thought-out clinical trial design strategy promise to overcome this sluggish advancement of LN treatment. Tektonidou et al. [25] offered the article in third place (23.20), which summarized a risk assessment model for ESRD in adult patients with LN by searching for cohort studies and clinical trials on ESRD from 1971 to 2015. Limitations in the effectiveness or accessibility of current treatment were identified through this model. The article in fourth place (22.99) was provided by Hanly et al. [26] By tracking nephritis outcomes in a multiethnic/racial SLE initial cohort, researchers found that despite the current standard of care, 38.3% of SLE patients initially developed LN, nephritis was linked to ESRD and death, and renal insufficiency was connected to a lower quality of life in terms of health. It is believed that the optimal treatment for LN requires further study. The article in fifth place (17.87) was provided by Arazi et al. [27] and revealed two chemokine receptors in LN, CXCR4 and CX3CR1, which suggest a potentially important role in cell trafficking. The article in sixth place (16.74) was submitted by Fanouriakis et al. [28] and provided revised consensus recommendations for the management of SLE, integrating evidence-based medicine and expert opinion. The article in seventh place (13.58), provided by Yu et al. [29], discusses several pathophysiological mechanisms contributing to LN and argues that fully understanding these mechanisms can provide reliable information for therapeutic approaches.

In addition to the reference, keywords can also relate to an area's major subject and essential elements. Using a co-occurrence evaluation of highly frequent keywords, three research directions for LN were identified (Fig. 6A): “the pathogenesis and manifestations of LN,” “the risk factors and methods of preventing LN,” “the treatment of LN," and “the classification and outcome of LN.” The evolution of high-frequency keywords is used to better comprehend the mutative trajectory of LN research themes (Fig. 6B). The colors of the keywords are determined by the average time the items occur; for example, red keywords typically come after blue keywords. Cluster 2 was where the majority of blue keywords first appeared early (green, focusing on treatment). In past years, Cluster 1 (red, focusing on pathogenesis and symptoms) and Cluster 3 (blue, focusing on classification and outcome) appear to include the majority of the red keywords. This indicates that after 2018, more research should focus on the pathogenesis, symptoms, categorization, and prognosis of LN.

The top 25 burst keywords between 2012 and 2022 were also identified. We particularly concentrate on those that will continue to explode into 2022, indicating that these are promising new areas of study. According to Fig. 7, certain terms, such as multitarget therapy, lupus erythematosus, international society, TMA, and pathway, are primarily related to the investigation of the therapy and mechanisms of LN.

LN is a heterogeneous disease with pathogenesis involving the immune dysregulation of B cells, T cells, etc. Traditional treatments include glucocorticoids and immunosuppressive agents, with limited therapeutic efficacy, necessitating the combination of other immunosuppressive agents targeting multiple aspects of the immune response, called multitarget therapy [30]. A recent randomized trial conducted at 26 renal centers in China showed that a multitargeted combination of MMF, tacrolimus(TAC) and corticosteroids for LN resulted in higher rates of complete remission and overall remission compared to IVCY [31]. Jia Fu et al. found that in addition to providing additional immunosuppression, combination therapy (prednisone, mycophenate, and TAC) protects the kidney through molecular pathways by inhibiting TLR7 expression [32]. A meta-analysis of six randomized controlled studies revealed that multitarget therapy had a better complete response rate than monotherapy. However, the multitarget treatment group had more infections and pneumonia cases than the monotherapy group [33]. Additional research is required to determine the effectiveness and safety of multitarget therapy in the broader population of LN patients.

TMA, a condition characterized by thrombocytopenia, microvascular hemolytic anemia, and renal failure, is one of the most prominent microangiopathies in LN. TMA can occur in LN patients with an incidence of 0.6–24.3% [34, 35]. Eculizumab is an effective therapeutic agent for patients with acute TMA, and Gillian M et al. concluded that 80% of patients taking eculizumab could achieve an event-free state of thrombotic microangiopathy based on the results of two 26-week phase II trials [36]. However, TMA still occurs in 20% of patients. Xiaopan Chen et al. s study proved that TMA, as an independent risk element for LN kidney failure, can seriously affect the prognosis of LN [37]. Therefore, specific diagnostic indicators should be determined, and effective treatment of LN should be conducted. Christine et al. demonstrated the significance of complement and acquired complement abnormalities in TMA and SLE. They demonstrated the roles of thrombomodulin, MCP/CD46, complement Factor H, complement Factor I, and complement Factor B in the development of TMA [38]. The prevalent “whole house” pattern (positive staining for IgG, IgM, IgA, C3, c1q) in LN and TMA monitored by Binsan Zhang et al. using immunofluorescence suggests that the pathogenesis of LN and TMA is influenced by LP and AP [39]. Establishing early warning signs for the identification of complement deficiency can help identify LN and TMA and improve outcomes.

### Basic information

This study employs bibliometric analysis to systematically and visually examine LN to identify its research status and research hotspots. The quantity and tendency of the annual publications were studied to gain insight into the progress of the discipline. The study's qualitative and quantitative analysis using CiteSpace and VOSviewer software shows that the annual production of scientific publications on LN, while trending downward in some periods over the past 11 years, has shown an overall upward trend and peaked in 2022 with 256 papers, indicating that LN has garnered growing interest from academics and experts.

Table 1 presents the productivity and contributions of different countries in LN. China (632, 30.43%), the United States (411, 19.79%), and Japan (118, 5.68%) are the top three productive countries, accounting for 55.90% of the total. However, when adjusting according to the population scale, South Korea came first with 1.29 articles for every million people. Notably, among the top ten countries, only China, Egypt and Brazil are developing countries. The variations in research output among countries may be due to differences in socioeconomic status, capacity for research, and population scale [40]. Furthermore, four of the top ten countries, including the United Kingdom (0.23), China (0.14), the United States (0.14), and Italy (0.10), had high centrality values greater than 0.1, suggesting their significant influence on LN research. In terms of institutional contributions (Table 2), the Ohio State University (USA) has the highest number of papers (74, 3.56%), while Shanghai Jiao Tong University ranks first with a centrality value of 0.27, indicating the strong influence of this institutions in the worldwide LN field. The top ten institutions were all from China or the United States, revealing the dominance of both countries in LN over the past 11 years. As illustrated in Fig. 2C, the close collaboration between countries and institutions is mainly concentrated in the United States and China, both of which have a high volume of publications. To promote prosperity in this field, exchanges and cooperation between these two countries and other countries, especially developing countries, should be enhanced further.

As demonstrated in Tables 3 and 4, Brad Rovin, having contributed 44 publications is the most prolific author. At the same time, Weening JJ is the most frequently cocited author with 809 cocitations. Half of the top ten authors possess a critical bridging function (centrality > 0.1). Weening JJ has remarkably secured the top spot in the list of cocited authors despite his limited publication record. This is because of the high impact of his articles [41]. Notably, Brad Rovin, affiliated with The Ohio State University Wexner Medical Center, has been actively involved in molecular expression research to establish a personalized and sustainable LN management framework for individual patient’s clinical and pathologic characteristics since 2017 [16, 42].

By analyzing the journals, Lupus was found to be the most active (267, 12.86%), followed by Frontiers Immunology (56, 2.70%) and Nephrology Dialysis Transplantation (50, 2.41%) (Table 5). Since there are a large number of LN-related papers in these periodicals, scholars interested in LN research can focus more on them. In terms of journal sources, the top ten most active journals in LN research were primarily from Western Europe, North America, and Oceania, highlighting the contribution of these regions to the field. Notably, even though Asia has produced 34.09% of the LN literature in the last 12 years, none of the journals were included in the top ten list, suggesting that LN-related journals in Asia must raise the calibre of their publications to encourage more writers to publish. Of the cocited journals (Table 6), Lupus was the most cited journal (5430), followed by Kidney International (3971), the Journal of the American Society Of Nephrology (3687), Arthritis And Rheumatism (3297) and the Journal of Immunology (2,867). Remarkably, Lupus is not only the journal with the highest number of publications but also far ahead of other journals in cocitations, providing evidence of its preeminence in the field of LN. Among the top ten cocited journals, six belong to the Q1 JCR category, underscoring the interest in high-impact publications in LN research. Furthermore, the fact that there is a 40% concordance rate between the top ten journals and cocited journals suggests that these journals are simultaneously striving to upgrade both the quantity and quality of research output in this field.

The number of citations, to a certain extent, reflected the impact of a work. The top ten most cited papers on lupus are listed in Table 7 which focus on recent research advances, classification and definition, treatment and management, and outcomes. The publication with the most citations was “Update on Lupus Nephritis” by Almaani et al. published in the Clinical Journal of the American Society of Nephrology in 2017. This review focuses on the current understanding of the disease epidemiology, genetics, pathogenesis, and treatment to establish a framework for the management of LN that targets patient-specific and patient-oriented maintenance of long-term renal function [16].

### Limitations

The study included several restrictions. First, restricting the search to titles may improve relevancy and accuracy, but some relevant articles will be missed unavoidably. Second, the WoSCC database was the only source of bibliometric data, eliminating other important databases and certain relevant studies. Third, we solely examined English-language texts, suggesting that non-English-speaking nations' contributions were sometimes missed. Finally, some newly released papers might not be included because of the delay.

## Conclusions

The bibliometric analysis of LN-related papers presented in this study is the first of its kind and offers insights into the quantity and caliber of research in this area. The findings show a rise in LN research worldwide during the last 11 years, with China and the United States dominating the area. The top three most productive institutions are the Ohio State University, the University of Peking and Shanghai Jiao Tong University. Brad Rovin is the most prolific and highly quoted author in this field, and Lupus is the journal with the most publications and cited publications. Importantly, contemporary studies concentrate on the keywords “voclosporin,” “biomarker,” and “nirp3 inflammasome” in the LN domain. Promising research directions for LN management include the pathogenic processes, prognostic outcomes, and management techniques. This bibliometric analysis thoroughly reviews the state and future directions in LN research. The results can guide future investment choices and research orientations as well as identify new partnership opportunities.

## Data Availability

The original contributions presented in the study are included in the article/supplementary material, and further inquiries can be directed to the corresponding authors.
